# Random Fiber Grating Characterization Based on OFDR and Transfer Matrix Method

**DOI:** 10.3390/s20216071

**Published:** 2020-10-26

**Authors:** Zichao Zhou, Chen Chen, Ping Lu, Stephen Mihailov, Liang Chen, Xiaoyi Bao

**Affiliations:** 1 Department of Physics, University of Ottawa, 25 Templeton Street, Ottawa, ON K1N 6N5, Canada; cchen340@uottawa.ca (C.C.); liang.chen@uottawa.ca (L.C.); 2 National Research Council Canada, Ottawa, ON K1A 0R6, Canada; Ping.Lu@nrc-cnrc.gc.ca (P.L.); Stephen.Mihailov@nrc-cnrc.gc.ca (S.M.)

**Keywords:** random fiber grating, degree of randomness, entropy, OFDR, transfer matrix method

## Abstract

Random fiber gratings (RFGs) have shown great potential applications in fiber sensing and random fiber lasers. However, a quantitative relationship between the degree of randomness of the RFG and its spectral response has never been analyzed. In this paper, two RFGs with different degrees of randomness are first characterized experimentally by optical frequency domain reflectometry (OFDR). Experimental results show that the high degree of randomness leads to low backscattering strength of the grating and strong strength fluctuations in the spatial domain. The local spectral response of the grating exhibits multiple peaks and a large peak wavelength variation range when its degree of randomness is high. The linewidth of its fine spectrum structures shows scaling behavior with the grating length. In order to find a quantitative relationship between the degree of randomness and spectrum property of RFG, entropy was introduced to describe the degree of randomness induced by period variation of the sub-grating. Simulation results showed that the average reflectivity of the RFG in dB scale decreased linearly with increased sub-grating entropy, when the measured wavelength range was smaller than the peak wavelength variation range of the sub-grating. The peak reflectivity of the RFG was determined by $\kappa^{2}L\Delta P$ (where κ is the coupling coefficient, L is the grating length, ΔP is period variation range of the sub-grating) rather than κL when ΔP is larger than 8 nm in the spatial domain. The experimental results agree well with the simulation results, which helps to optimize the RFG manufacturing processes for future applications in random fiber lasers and sensors.

## 1. Introduction

Over the past few years, random fiber gratings (RFGs) have drawn a great deal of interest in the field of random fiber lasers and optical fiber sensing [1,2,3,4,5]. By introducing refractive index modulations with random periods in the fiber core, RFGs can mimic Rayleigh scattering in the fiber with a broadband reflection spectrum. Compared with the fiber’s intrinsic inhomogeneity, RFGs introduce much stronger random refractive index variations, greatly enhancing light backscattering over a short fiber length. Various kinds of RFGs have been made for applications in sensors and random fiber lasers. A multi-parameter fiber optic sensor was demonstrated based on a femtosecond laser micro-machined random grating, which was able to measure temperature, axial strain, and surrounding refractive index simultaneously [6]. By using scattering from an RFG instead of the traditional Rayleigh scattering, the sensing accuracy and spatial resolution of optical frequency domain reflectometry (OFDR) was greatly enhanced [7,8]. RFGs can also provide distributed random feedback for random fiber lasers to improve their performance. A narrow linewidth low-intensity noise tunable fiber laser with Erbium-doped gain was realized by introducing randomly spaced index modulations on a standard telecommunication single-mode fiber [9]. A multi-wavelength Brillouin random fiber laser could be achieved via distributed feedback from a RFG [10]. Based on a random coherent feedback mechanism, a long random fiber Bragg grating was demonstrated for a coherent Raman random fiber laser [11]. Lévy intensity statistics were found in a one-dimensional erbium-doped random fiber laser which was based on an RFG [12]. Compared to the distributed feedback from Rayleigh scattering, the enhanced random reflections from an RFG could tolerate environmental perturbations and reduce resonating modes in Brillouin random fiber lasers, leading to reduced lasing thresholds and low intensity and frequency noises [13,14]. Based on the random fiber laser, a highly sensitive ultrasound sensor was realized where an RFG provided distributed random feedback as well as acting as the ultrasound sensing head [15,16]. Since RFGs have numerous applications, it is important to characterize the RFG in order to enhance the manufacturing processes and to elevate its performance. Experimentally, OFDR has proven to be excellent for characterizing regular fiber Bragg gratings (FBGs) because of its ultra-high spatial resolution [17]. In the OFDR technique, the beat signal between a linear frequency swept laser and its time-delayed replica from the device under test was recorded. By doing spectral analysis of the beat signal, the reflection intensity of the device under test versus the distance map could be obtained. The spatial resolution of OFDR, which is determined by the frequency sweeping range of the tunable laser, can be as precise as several micrometers. The spectral response of the device under test can be retrieved by OFDR when an appropriate window is applied to the corresponding location in the spatial domain. Theoretically, the transfer matrix method [18] can simulate the grating properties by calculating the input and output fields over a short section of the sub-grating. The random parameters can be added to these sub-gratings in the simulation in order to study how the degree of randomness affects the grating’s properties. However, to the best of our knowledge, the degree of randomness of a RFG has never been quantified. There is still no quantitative relationship between the degree of randomness and the grating properties up to now.

In this paper, the reflectivity and spectral response of RFGs were characterized by the OFDR technique experimentally and simulated by the transfer matrix method theoretically. In the experiment, two RFGs with different degrees of randomness resulting from different sub-gratings period variation ranges were characterized by the OFDR technique. This characterization provided a qualitative description of the relationship between the degree of randomness and the performance of the RFG. A high degree of randomness leads to low average reflectivity and strong reflectivity fluctuations of the RFG in the spatial domain. Local spectral responses of RFGs with a high degree of randomness exhibit multiple peaks with large peak wavelength ranges. Furthermore, the linewidth of the fine spectral structure of RFGs shows scaling behavior with grating length. In simulation, entropy can describe the degree of randomness of the RFG mathematically. We find a linear relationship between the sub-grating’s entropy and the average reflectivity of the RFG in dB scale. The averaged reflectivity of the RFG is determined by the sub-grating’s entropy rather than the entropy of the total RFG, hence the localized grating features dominate the grating spectrum. The experimental results agreed well with the simulation results, which provide the quantitative relationship between the degree of randomness and the spectral property of the RFG that is important for the application of RFGs.

## 2. Experimental Characterization of RFG

**Figure 1:**
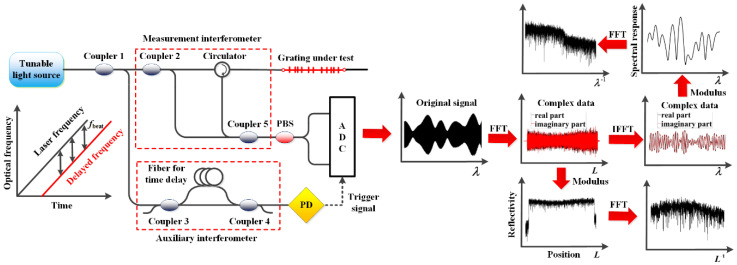
Experiment setup for RFG characterization and data processing method.

The RFG characterization setup is shown in Figure 1. The optical system comprised a tunable laser source, an auxiliary interferometer, a measurement interferometer, and an optical receiver system. With the tunable laser sweeping its output frequency linearly, an optical beat wave is generated in the auxiliary interferometer, which is used to trigger the sampling of the signal from the measurement interferometer at equal intervals of wavelength. In the measurement interferometer, the tunable light is launched into two arms: one arm is the reference arm and the other arm is connected to the grating under test. Light reflected from different positions along the grating generate different beat frequencies with the light in the reference arm. The beat signal is then collected by the photodetector and sent to the analog to digital conversion (ADC) system, which is the original real-valued signal that we get from the OFDR setup in the wavelength domain. The data processing method used to characterize the RFG is shown on the right side of Figure 1. First, by doing a fast Fourier transform (FFT) of the original real-valued signal in the wavelength domain, a complex data set containing the amplitude and phase of the reflectivity can be calculated in the spatial domain. The reflectivity of RFG at different positions is then obtained by taking the modulus of the complex signal in the spatial domain. In order to study the reflectivity characteristics of the RFG along the spatial domain, an FFT is also applied to the modulus of the reflectivity for further analysis. At the same time, an appropriate window is applied to the complex data in the spatial domain with an inverse FFT (IFFT) to transform the signal back to the wavelength domain. The restored data in the wavelength domain is also a complex data set with its modulus representing the local spectral response of the selected locations of the RFG. The fine structures of the spectral response of the RFG is then analyzed with another FFT calculation.

In the experiment, two RFGs with different sub-grating period variation ranges were characterized. Both of the RFGs were inscribed by a femtosecond laser with the same pulse energy and comprised of a large number of uniform sub-gratings. The length of each sub-grating was 0.025 mm, and the total length of each grating was 50 mm. The difference was that the period of the sub-gratings in the first RFG were randomly distributed between 0.5328 and 0.5436 μm with 10.8 nm maximum difference, which is much smaller than optical wavelength of 1.5 μm. In the second RFG, the period of each sub-gratings were randomly distributed from 0 to 2.5 μm [8]. The different scale of random period fluctuation in the RFG gves different degrees of randomness within the fiber medium. The degree of randomness describes the disorder of the system. For example, the RFG with a large sub-grating’s period variation range has a relatively high degree of randomness and vice versa. In the following, we named the RFG with a high degree of randomness as a “high disordered RFG” and the other as a “low disordered RFG” for the convenience of description.

### 2.1. Reflectivity Characterization in Position Domain

**Figure 2:**
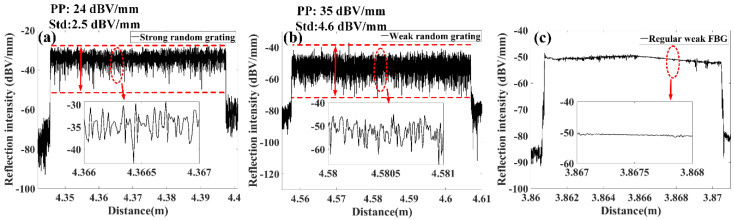
Reflectivity versus distance of (**a**) low disordered RFG, (**b**) high disordered RFG, and (**c**) regular weak FBG.

In order to characterize the reflectivity induced by the random period change of sub-gratings, the backscattering level is first measured by OFDR at different locations along the RFG, as shown in Figure 2. Figure 2a,b show the backscattering strength of high and low disordered RFGs, respectively. The average backscattering strength per unit length of the low disordered RFG (−34 dBV/mm) is higher than that of high disordered RFG (−54 dBV/mm) even though the refractive index modulation and grating length for both types of RFG are identical. In other words, a high degree of randomness reduces the reflection intensity of the RFG. The reason behind the different reflectivity is that with a lower degree of randomness and smaller sub-gratings period variation (<10.8 nm) in the low disordered RFG, the random phase change of the backscattered light in a local region is less stochastic than that in the high disordered RFG with larger grating period fluctuation (<2.5 μm, which is larger than optical wavelength around 1.5 μm). This leads to partially correlated phase superposition in the low disordered RFG and uncorrelated phase superposition in high disordered RFG. As a comparison, the reflectivity of a regular uniform weak FBG is also measured. It can be seen in Figure 2c that the reflection intensity of the regular weak FBG in the spatial domain is quite smooth, except for some small fluctuation at particular positions, which are caused by fabrication flaws. With an 8 μm spatial resolution of the OFDR setup, the measured data can be considered as reflection intensity from discrete reflectors separated by 8 μm along the RFG. The random period change of the sub-gratings would lead to reflection intensity fluctuations of these reflectors. The peak to peak value and standard deviation of these reflector’s reflection intensity in the high disordered RFG are 35 dBV/mm and 4.6 dBV/mm, respectively. These values are much larger than the corresponding value (24 dBV/mm and 2.5 dBV/mm) for the low disordered RFG. Though higher degrees of randomness in the high disordered RFG give rise to lower average reflection strength of each reflector, the local reflection strength fluctuation is much stronger in the high disordered RFG than that in the low disordered RFG.

**Figure 3:**
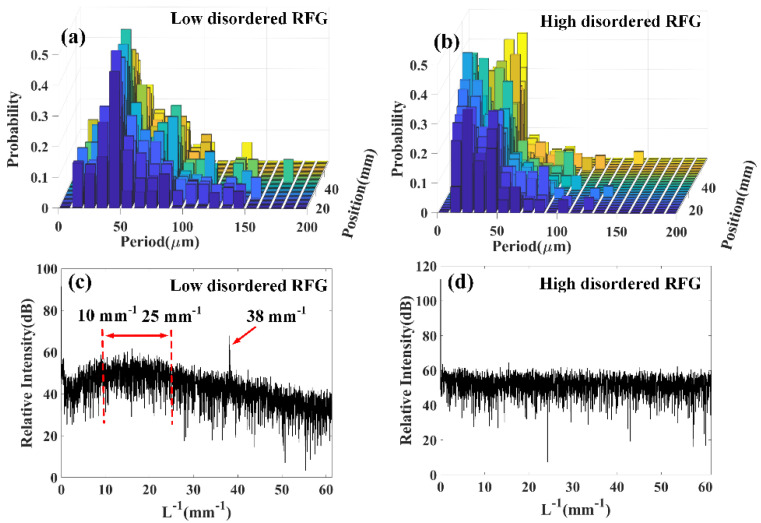
(**a**) Histogram of the reflection intensity fluctuation period at every 1 mm region of the low disordered RFG. (**b**) Histogram of the reflection intensity fluctuation period at every 1 mm region of the high disordered RFG. (**c**) Fourier transform of the reflection intensity of the low disordered RFG. (**d**) Fourier transform of the reflection intensity of the high disordered RFG.

To further illustrate the influence of the degree of randomness to the grating’s reflectivity property, a probability histogram of the reflection intensity fluctuation period at every 1 mm range of the RFG was plotted. The reflection intensity fluctuation period here refers to the distance between two adjacent local minima points in Figure 2. It can be seen from Figure 3a that for the low disordered RFG, the fluctuation period was most likely located at 50 μm. In contrast, Figure 3b shows that the fluctuation period in the high disordered RFG is most likely located between 10 μm and 50 μm. Because the spatial resolution of the OFDR setup in the experiment was 8 μm, each point in Figure 2 represents the light reflection strength in the local area. The histograms of Figure 3a,b indicates that the low disordered RFG showed periodical reflectivity features in the spatial domain, while the high disordered RFG exhibited stochastic reflection intensity distribution. The difference was also caused by the partially correlated phase superposition in the low disordered RFG and the stochastic phase superposition in the high disordered RFG. By selecting the RFG part in Figure 2 and doing a Fourier transform of the modulus of the reflection intensity, the intensity fluctuation frequency was calculated, which is shown in Figure 3c,d. It can be seen that the relative intensity at each fluctuation frequency in the high disordered RFG was quite flat, indicating that there is no particular fluctuation frequency and the reflection intensity at different positions was totally randomly distributed. In contrast, the fluctuation frequency of the low disordered RFG showed an envelope where the maximum value was located between 10 mm^−1^ and 25 mm^−1^, corresponding to the approximately 20 oscillations in the 1 mm range of the inset figure in Figure 2a. The periodic intensity fluctuation feature in Figure 2a resulted from the relatively small period fluctuation range of the sub-gratings in the low disordered RFG. It is interesting to note that there was a small sharp peak at 38 mm^−1^ in Figure 3c, although it is hard for us to directly see the 38 oscillations of intensity fluctuation in the 1 mm range. The 38 mm^−1^ oscillation frequency in the spatial domain comes from the inverse of the sub-grating’s length (~0.025 mm). The sharp peak further proves that low degree of randomness in a fiber grating would lead to periodic reflection intensity features in the spatial domain.

### 2.2. Local Spectral Response Characterization

**Figure 4:**
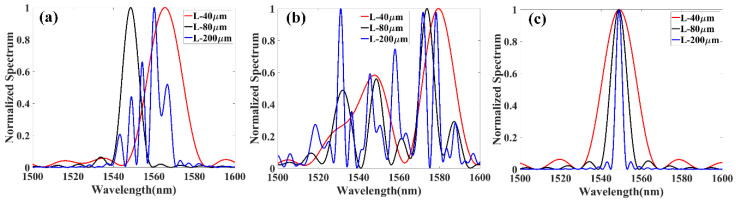
Local spectral response of (**a**) low disordered RFG, (**b**) high disordered RFG, (**c**) regular weak FBG at different lengths.

Thanks to the high spatial resolution of the OFDR technique, the local spectral response of the RFG could be characterized. The influence of the grating length to the local spectral response property was first studied both in low and high disordered RFGs. It can be seen from Figure 4a that when the interrogation length was chosen to be 40 μm and 80 μm, the local spectral response of the low disordered RFG only had one dominant peak, indicating that the refractive index fluctuation under the length of 80 μm was not random enough to create multiple reflection peaks in the spectral domain. When the interrogation length was increased to 200 μm, multiple reflection peaks started to appear due to the period change of the sub-gratings in the local region. The local spectral response in the high disordered RFG in Figure 4b revealed different properties. When the interrogation length of high disordered RFG was chosen to be as small as 40 μm, multiple peaks still appeared in the local spectral response and the peak number increased as the interrogation length elongated. The local spectrum of the high disordered RFG showed a larger number of peaks than the low disordered RFG, which was caused by the relatively high degree of randomness in the high disordered RFG. As a comparison, the local spectral response of a regular weak FBG was also measured. It can be seen that for regular uniform FBG, there is only one dominant reflection peak regardless of the grating’s length, illustrating that the higher degree of randomness would induce more reflection peaks in the spectral domain.

**Figure 5:**
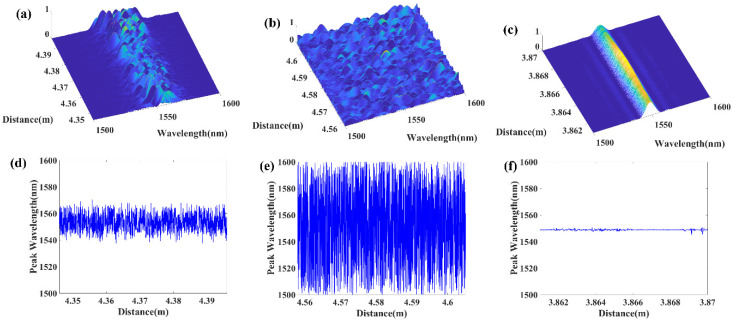
Distributed local spectral response of (**a**) the low disordered RFG, (**b**) the high disordered RFG, (**c**) a regular weak FBG at different positions. And the peak wavelength of the local spectral response of (**d**) the low disordered RFG, (**e**) the high disordered RFG, and (**f**) the regular weak FBG at different positions.

Figure 5 shows the distributed local spectral response of the low disordered RFG, high disordered RFG and a regular uniform FBG. The interrogation length was chosen to be 40 μm. The top three figures show their three-dimensional local spectral response at every position, and the bottom three figures are the corresponding peak wavelength value in the spatial domain. It can be seen that in the regular uniform FBG, the peak wavelength of the local spectral response was always located at 1550 nm, except for some small deviation at a particular position. The standard deviation of the distributed peak wavelength in regular uniform FBG was 0.1 nm. The reflection strength in the middle part was stronger than both sides of the measured uniform FBG, which also agreed with the reflectivity changing trend at different positions, shown in Figure 2c. For the low disordered RFG, the peak wavelength of the spectral response at each local section randomly changes from 1540 nm to 1570 nm, with a standard deviation of 6.72 nm. In contrast, the corresponding peak wavelength of each local section in the high disordered RFG covered the total wavelength range from 1500 nm to 1600 nm, and the standard deviation is 26.65 nm, which was 4 times larger than the corresponding value in the low disordered RFG. Both the low and high disordered RFGs had a much larger standard deviation of distributed peak wavelength than regular uniform FBG. The local spectral response showed that the higher degree of randomness could provide richer reflection peaks for the RFG in the spectral domain and resulted in larger peak wavelength fluctuation in the grating’s spatial domain.

### 2.3. Linewidth Scaling Behavior

**Figure 6:**
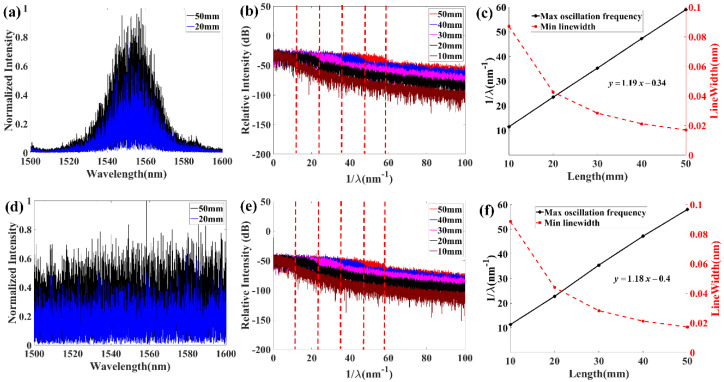
(**a**) Reflection spectrum of the low disordered RFG measured by OFDR. (**b**) Fourier transform of the reflection spectrum of the low disordered RFG. (**c**) Relationship between the maximum spectrum oscillation frequency and grating length. (**d**) Reflection spectrum of the high disordered RFG measured by OFDR. (**e**) Fourier transform of the reflection spectrum of high disordered RFG. (**f**) Relationship between the maximum spectrum oscillation frequency and grating length.

When a laser beam propagates in a random medium, its lightwave nature leads to many interesting phenomena. For example, the well-known Anderson localization in a random medium is now understood to be a universal wave phenomenon due to multi-scattering and coherent interference [19,20]. Shapira and Fischer studied the light localization effect in an RFG array in single-mode fiber [18]. They showed that for a stack of N sub-gratings with randomly varying intervals, the transmitted intensity decays exponentially rather than linearly with the number of sub-gratings N. Actually, due to light multi-scattering and coherent interference, the reflection spectrum from a random medium shows narrow linewidth peaks in the spectral domain. Those narrow linewidth peaks act as a narrow linewidth filter in the application of random fiber lasers [7,11], which is an important feature of RFG. Figure 6a,d shows that the high disordered RFG had a very broadband reflection spectrum envelop while the low disordered RFG had stronger reflection at particular wavelengths from 1520 nm to 1580 nm. Both high and low disordered RFGs have many fine structures in the reflection spectrum. An important feature of those fine structures is the linewidth of each small peak, which could be analyzed by doing Fourier transform to the reflection spectrum. Because lightwave interference and multi-scattering are affected by the length of RFG, reflection spectra for different RFG lengths are studied. Figure 6b,e are the Fourier transforms of the reflection spectrum in Figure 6a,d, respectively. It can be seen that there is always a drop point after the FFT calculation, which corresponds to the maximum oscillation frequency of the reflection spectrum. When the length of the RFG is 50 mm, the maximum spectrum oscillation frequency is around 60 nm^−1^ for both low and high disordered RFGs. The maximum spectrum oscillation frequency increases linearly with increased RFG length, indicating that the minimum linewidth of the fine spectrum structures decrease inversely proportional to the grating length, as shown in Figure 6c,f. The maximum spectrum oscillation frequency was not affected by the degree of randomness, as low and high disordered RFGs showed similar behavior in Figure 6c,f. A question here is whether the OFDR setup had enough spectral resolution to characterize the fine structures of RFG. In [21], the spectral resolution of the OFDR setup is as small as 3.94 pm when the spatial resolution is set at 3.85 mm, corresponding to the maximum measurable spectrum oscillation frequency (0.5/3.94 pm) of 147 nm^−1^. The 50 mm length of the RFG in our experiment is much longer than 3.85 mm and the maximum 60 nm^−1^ spectrum oscillation frequency is also smaller than 147 nm^−1^. Therefore, the OFDR setup has adequate spectral resolution to characterize the fine spectrum structures of the RFG.

## 3. Theoretical Simulation of RFG

In the experiment, two different disordered RFGs were characterized, which gave a qualitative description about the influence of the degree of randomness to the gratings’ property. In order to find the quantitative relationship between the degree of randomness and reflectivity of the RFG, the concept of entropy was introduced to describe the degree of randomness of the RFG. The period of each sub-grating can be regarded as a continuous random variable with uniform distribution. The probability density function of a random variable ranges from a to b with uniform distribution is:

(1) $$p(x)=\left\{\begin{array}{c} \frac{1}{b-a},a<x<b\\ 0,otherwise \end{array}\right\}$$

**Figure 7:**
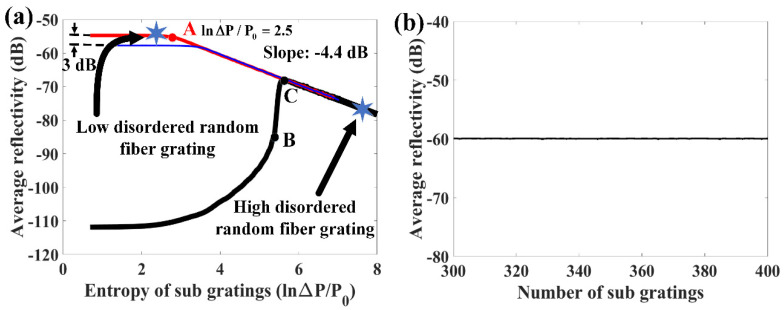
(**a**) Relationship between the average reflectivity of RFG and entropy of sub-gratings (the red curve represent the median value of the sub-grating’s period were 0.5328 μm, and calculated wavelength ranges from 1500 nm to 1600 nm. The blue curve represents the median value of the sub-grating’s period are 0.5328 μm, and calculated wavelength ranges from 1450 nm to 1650 nm. The black curve represents the median value of the sub-grating’s period are 0.6500 μm and calculated wavelength ranges from 1500 nm to 1600 nm); (**b**) Relationship between the average reflectivity of the RFG and number of sub-gratings.

Because the RFG is comprised of many sub-gratings that are independent of each other, the entropy of the whole RFG can be defined as [22]:

(2) $$H(X_{1},X_{2}\cdot \cdot \cdot X_{N})=N\cdot H(X)=-N\cdot \int p(x)\ln p(x)dx=N\cdot \ln\Delta P$$

where ΔP is the period variation range of all sub-gratings, N is the total number of sub-gratings, H(X) is the entropy of a sub-grating. The reflection spectrum of the RFG is simulated based on the transfer matrix method [23]. In the transfer matrix method, propagation of light characterized by the transfer matrix relates the amplitudes of the forward and backward propagating waves of each sub-grating on the right-hand side with those on the left-hand side:

(3) $$\left[\begin{array}{c} a_{n}^{+}\\ a_{n}^{-} \end{array}\right]=M_{n}\left[\begin{array}{c} a_{n-1}^{+}\\ a_{n-1}^{-} \end{array}\right]$$

where $a_{n}^{+}$ and $a_{n-1}^{-}$ are the transmission and reflection field of the $n_{th}$ sub-grating, respectively. $M_{n}$ is the transfer matrix of $n_{th}$ sub-grating. The transfer matrix of the whole RFG can be written as the product of the transfer matrix of each sub-grating. From the coupled-wave equation of the counter-propagating waves, the transfer matrix of a single uniform sub-grating can be written as:

(4) $$M_{n}=\left(\begin{array}{cc} \cosh(SL_{0})-i\frac{\Delta \beta }{S}\sinh(SL_{0}) & -i\frac{\kappa }{S}\sinh(SL_{0})\\ i\frac{\kappa }{S}\sinh(SL_{0}) & \cosh(SL)+i\frac{\Delta \beta }{S}\sinh(SL_{0}) \end{array}\right)$$

where $L_{0}$ is the length of each sub-grating. κ is the coupling coefficient between the counter-propagating beams in the gratings, which is determined by the refractive index modulation, Δβ=β−π/Λ is the wave-number deviation from the Bragg wavelength, Λ is the grating period, and $S={\left({\left|\kappa \right|}^{2}-\Delta \beta^{2}\right)}^{1/2}$. The relationship between the entropy of the sub-grating and mean reflectivity of the RFG is simulated first, as shown in Figure 7a. The main simulation parameters in Figure 7a are as follows. The refractive index modulation is $10^{-6}$, and every 50 uniform index modulation periods form a sub-grating, and the total number of sub-gratings is 380. The mean reflectivity is calculated by averaging the reflectivity of the RFG in the spectral domain. The period variation range is divided by a constant $P_{0}$($P_{0}=1\;\mathrm{nm}$) for normalization, thus the entropy of sub-grating is expressed as $\ln\Delta P/P_{0}$. The red curve is the result when the median value of sub-grating’s period is 0.5382 μm (corresponding to the central wavelength of 1550 nm) and the black curve is the result when the median value of sub-grating’s period is 0.6500 μm (corresponding to the central wavelength of 1872 nm). The red curve shows that the mean reflectivity of the RFG remains almost constant when the sub-grating’s entropy is smaller than 2.5 (before point A). This is similar to the conserved transmittance area theorem that is proved in [24], which claims that the total area under the curve of the transmittance in the reciprocal wave vector space remains constant regardless of the amount of disorder. However, when the grating’s period variation range continues to increase after point A, the reflectivity in dB scale decreases linearly with the increase of the sub-grating’s entropy. This is because the wavelength range in our simulation only covers from 1500 nm to 1600 nm. With increasing entropy of the sub-grating, the reflected energy is dispersed from the central wavelength to other wavelengths. If the dispersed wavelength range is smaller than the calculated wavelength range in the simulation (or wavelength range used for measurement in the experiment), the total reflectivity of the RFG remains constant. When the sub-grating’s entropy is large enough, the dispersed wavelength range would exceed our measured wavelength range, and the reflectivity of RFG at a fixed wavelength range would decrease exponentially as the sub-grating entropy increases. The two stars on the curve represent the two RFGs that were measured in the experiment by the OFDR setup. The period variation of the low disordered RFG in the experiment was 10.8 nm, corresponding to 2.38 of sub-grating’s entropy ($\ln\Delta P/P_{0}\approx 2.38$). The period variation of the high disordered RFG was 2.5 μm, corresponding to 7.82 of sub-grating’s entropy ($\ln\Delta P/P_{0}\approx 7.82$). The simulated average reflectivity of the high disordered RFG is 22 dB lower than that of the low disordered RFG, which is very close to the experiment result (~20 dB). Since the simulated random period of the sub-gratings is assumed to have a uniform distribution, the entropy of the high disordered RFG in the experiment was a little overvalued as the random period of the sub-gratings was more likely to be focused on a particular region. Considering this factor, the simulation results agreed well with our experimental results. The wavelength range is selected to be from 1500 nm to 1600 nm because the wavelength range of the tunable laser in the experiment is from 1500 nm to 1600 nm. A larger wavelength calculation range does not affect the mean reflectivity changing trend of the RFG. For example, the blue curve in Figure 7a represents that the calculated wavelength in simulation ranges from 1450 nm to 1650 nm. Since the wavelength range of the blue curve is two times larger than that of the red curve, the mean reflectivity in the blue curve is 3 dB smaller than that in the red curve when the entropy of sub grating is small, which means the total reflectivity of the RFG in these two cases is same. When the entropy of the sub grating is large, the blue curve also shows that the average reflectivity of the RFG in dB scale decreased linearly as the increase of the entropy of sub gratings. In order to study the reflectivity of the RFG that is out of the central wavelength range of the sub-grating, the median value of the sub-grating’s period is set at 0.6500 μm, as shown of the black curve in Figure 7b. When the sub-grating’s period variation range is smaller than 0.0944 μm (before point B), the total reflectivity of RFG increases slowly as the increase of sub-grating’s entropy. The wavelength from 1500 nm to 1600 nm located at the bandpass region of a uniform FBG with a grating period of 0.6500 μm, which is a result of the destructive interference of light. If small-period variation is introduced to the sub-grating, the destructive interference would be disturbed, leading to an increase of reflection intensity. When the sub-grating’s period variation range is between 0.0944 μm and 0.1292 μm (between point B and point C), the total reflectivity of RFG increases dramatically as the increase of sub-grating’s entropy. The dramatic increase relies on that the central wavelength of the sub-grating starts to cover the wavelength range from 1500 nm to 1600 nm when the sub-grating’s period variation range is larger than 0.0944 μm. If the sub-grating’s entropy continues to increase (after point C), the reflectivity of the RFG decreases exponentially as the increase of the sub-grating’s entropy after the central wavelength of the sub-grating totally covers the wavelength range from 1500 nm to 1600 nm. In order to study whether the average reflectivity of the RFG is dependent on the number of sub-gratings or the total entropy of the RFG, the number of sub-gratings is changed from 300 to 400 while the total length of the RFG was fixed (10 mm). Due to the requirement of the slowly varying approximation in the transfer matrix method, the number of the periods in each sub grating should be a suitably large number. When the number of sub gratings is chosen to be 400, the period number of each sub grating is 47, which is large enough to ensure accurate simulation result [23]. Figure 7b shows that the average reflectivity of the 10 mm long RFG is independent of the number of sub-gratings, which implies that the reflectivity of the RFG is determined by the entropy of each sub-grating rather than the total entropy of the RFG.

**Figure 8:**
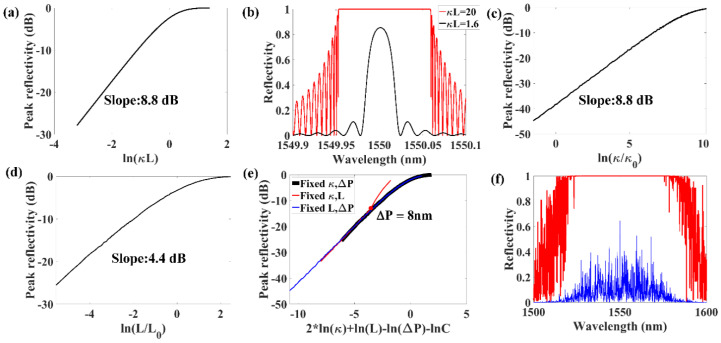
(**a**) Relationship between peak reflectivity of a FBG and lnκL. (**b**) Spectrum of a regular FBG when κL=1.6 (black curve) and κL=20 (red curve). (**c**) Relationship between peak reflectivity of a RFG and $\ln L/L_{0}$. (**d**) Relationship between peak reflectivity of a RFG and $\ln\kappa /\kappa_{0}$. (**e**) Relationship between the peak reflectivity of an RFG and 2*lnκ+lnL−lnΔP−lnC (black curve: fixed κ,ΔP; red curve: fixed κ,L; blue curve: fixed L,ΔP). (**f**) Spectrum of a RFG when $\kappa^{2}L\Delta P>>C$ (red curve) and $\kappa^{2}L\Delta P<<C$ (blue curve).

The experimental results show that the linewidth of the fine spectrum structures of the RFG decrease inversely proportional to the increase of the RFG length, which was similar to the linewidth of a regular FBG ($\Delta \lambda \approx \frac{\lambda^{2}}{2n_{eff}L}$) when κL<<π. Otherwise, the linewidth of a regular FBG was proportional to the coupling constant ($\Delta \lambda \approx \frac{\lambda^{2}\kappa }{\pi n_{eff}}$) if κL>>π [21]. The relationship between the peak reflectivity of a regular FBG and ln(κL) show different characteristics in these two conditions. When κL<<π, the peak reflectivity of an FBG in dB scale increases linearly with the increase of ln(κL). In contrast, the peak reflectivity of an FBG is close to 100% if κL>>π, as shown in Figure 8a. Figure 8b shows the spectrum of an FBG at different conditions when κL=1.6 and κL=20. When random period variation of the sub-gratings is introduced into the grating, the peak reflectivity of the RFG is reduced and the criteria that κL<<π or κL>>π can be used to estimate the grating properties is no longer valid. In order to find the proper criteria that can estimate the properties of the RFG, the peak reflectivity of the RFG is shown in Figure 8c–e. There is a linear relationship between the peak reflectivity of the RFG and $\ln\kappa /\kappa_{0}$ ($\kappa_{0}$ is a constant) with a slope of 8.8 dB when κ is relatively small, as shown in Figure 8c. Similarly, Figure 8d shows that the relationship between the peak reflectivity and $\ln L/L_{0}$ ($L_{0}$ is a constant) is also close to linear with a slope of 4.4 dB when L is relatively small. According to the simulation results in Figure 8c,d and Figure 7a, we concluded that the peak reflectivity of the RFG was determined by $\kappa^{2}L\Delta P$ rather than κL, as the slope of the linear part in Figure 8d was two times the slope of the linear part in Figure 7a and Figure 8c. Figure 8e shows the relationship between the peak reflectivity of the RFG and 2*lnκ+lnL−lnΔP−lnC, where C was a dimensionless constant ($C=10^{-5}$). Since there were three variables κ,L and ΔP, there are three curves in Figure 8e, which is plotted by changing one of the variables among them and keeping the other two variables fixed. These three curves were overlapped with each other except when ΔP was smaller than 8 nm. The overlapping portion indicates that the peak reflectivity of the RFG was determined by $\kappa^{2}L\Delta P$ when the random period variation ΔP was larger than 8 nm. When ΔP was smaller than 8 nm, the peak reflectivity property of the structure is similar to a regular FBG rather than a RFG. Figure 8f shows two typical spectral shapes of RFGs when $\kappa^{2}L\Delta P>>C$ (red curve) and $\kappa^{2}L\Delta P<<C$ (blue curve).

## 4. Conclusions

In this paper, the reflectivity and spectral properties of RFGs were characterized by the OFDR technique and simulated based on the transfer matrix method. First, by comparing different characteristics of low and high disordered RFGs, influences of the degree of randomness to the grating’s backscattering strength and spectral response properties are qualitatively analyzed experimentally. Higher degrees of randomness in the RFG lead to lower light backscattering intensity with larger intensity fluctuations in the spatial domain. For low disordered RFGs, the low degree of randomness results in a specific intensity fluctuation period. For the local spectral response, a high degree of randomness leads to rich fine structures and a larger peak wavelength fluctuation range. The linewidths of the fine spectral features of the RFG shows a scaling behavior with the grating’s length. The concept of entropy is introduced to describe the period variation of the RFG in order to establish the quantitative relationship between the grating’s degree of randomness and its spectral properties. Simulation results show that the average reflectivity of a RFG is dependent on the sub-grating’s entropy rather than the entropy of the total RFG. The average reflectivity of RFGs in dB scale decreases linearly as the increase of the sub-grating’s entropy when the wavelength range for measurement is smaller than sub-grating’s central wavelength fluctuation range. The peak reflectivity of the RFGs are determined by $\kappa^{2}L\Delta P$ rather than κL, which provides a new valid criteria to estimate the spectrum properties of the RFGs. The experimental characterization results agree well with the simulation results, which provide us a better way to understand the detailed structure of a RFG, and also can help us to optimize the RFG manufacturing processes for future applications such as random fiber lasers and sensors.
